# Healed by our inner fish?

**DOI:** 10.18632/oncotarget.4594

**Published:** 2015-06-23

**Authors:** Philipp Niethammer

**Affiliations:** Cell Biology Program, Memorial Sloan Kettering Cancer Center, New York, NY, USA

The “gifts” passed down to us by our ancestors are sometimes more, sometimes less flattering - mom's skeptical spirit, dad's sense of duty, but then also: granddad's unfavorably large nose. Traits can help us, or stand in our way depending on how well they are adapted to (and liked by) the people, the environment around us. While the purpose of grandpa's nose remains puzzling, some inherited traits are unambiguously helpful. For example, our body's intriguing ability to avoid infections after we bite our tongue. Our mouth, and digestive tract environment is a rich biotope for microorganisms. Any epithelial damage to the linings of our digestive tract exposes the inside of our tissues to an army of malicious invaders. Although such damage occurs frequently during normal digestive activity (and may be enforced by certain epithelial diseases), it rarely causes infection unless we already suffer from serious immunodeficiency. The mucosal surfaces of our body cavities, unlike our “dry” epidermis, are all covered by liquid. They heal faster and with less inflammation and scarring compared to our outside shell [1–3]. Rapid epithelial healing is one of the most primitive and effective ways to keep pathogens out of our body. Although the correlation between the presence of a liquid layer and rapid, i.e., “privileged,” healing is conspicuous, causal connections between these two concepts have been little investigated, apparently for all the right reasons: dry epidermal wounds also heal, thus a liquid layer cannot be so important. However, this thinking somewhat neglects the evolutionary history of epithelial surfaces. Liquid covered epithelia are the more ancient barrier structures and constitute the largest part of our total surface area. By contrast, healing in the absence of environmental liquid is an evolutionary relatively new invention, which presumably evolved together with reptile life on land. Did the outer parts of our epithelial surfaces simply had to “re-learn” healing without liquid, and how may environmental liquids enhance healing?

Privileged healing is best studied in animals that still live in the environment where this mechanism once evolved: water. Over the last decades, a couple of studies in aquatic organisms have suggested a crucial contribution of the external liquid environment to epithelial healing [4]. One convenient laboratory model is to injure the tail fin of a larval zebrafish. This results in rapid recruitment of leukocytes and wound closure, followed by slower regeneration. Although this response has been long known, it has remained unclear how the tissue detects the wound [4]. Zebrafish are freshwater animals that live in a low osmolarity solution. Upon epithelial breaching, environmental liquid enters the fish tissue to dilute out interstitial osmolytes, which leads to local cell swelling. This swelling appears to play a central role in wound detection; if it is blocked by immersing the fish within a medium that is adjusted to the osmolarity of interstitial fluid, the wound is detected much less well, and healing and leukocyte recruitment is delayed [5,6].

**Figure 1:**
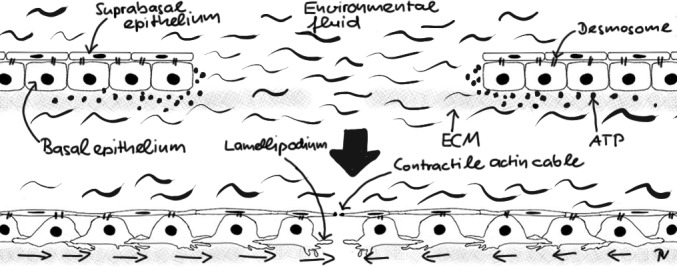
Simplified cartoon scheme of rapid healing response in larval zebrafish tail fin epithelium

The larval zebrafish tail fin skin is a simple stratified epithelium (Figure 1). Tight junctions in-between suprabasal cells provide water impermeability to the fish, desmosomes connect the suprabasal, and basal epithelial layers, and integrins tug the basal epithelial layer onto a basal lamina. Upon injury in fresh water, basal epithelial cells develop lamellipodia and start sliding on the basal lamina toward the wound dragging the suprabasal cells along with them [5]. Simultaneously, the suprabasal cells at the wound margin develop a contractile actin cable (“purse string”) that pulls on the sliding tissue. Basal cell migration is triggered by osmotically induced ATP release at the wound site. ATP has been implicated in healing of epithelial monolayers in cell culture through purinergic receptor activation. Although swelling induced ATP release has been well described in vitro, the mechanisms that lead to secretion and recognition of ATP in vivo still need to be clarified. Suprabasal actin cable contraction is operant even after isotonic injury, when basal epithelial cell migration is suppressed. Wound margin contraction alone, however, is barely strong enough to overcome the frictional forces that glue the basal epithelial layer onto the basal lamina. If not assisted by basal cell sliding, the purse-string can only close very tiny breaches on its own. In other words, environmental liquid exposure allows contractile and migratory wound closure mechanisms to synergize, which accelerates healing. Environmentally induced cell swelling also activates enzymes that make inflammatory lipid mediators [6] to rapidly call leukocytes to the wound. Thus, in zebrafish larvae, environmental liquid is a master mediator of both, fast antimicrobial as well as healing responses after epithelial wounding. Whether the luminal liquid layers of mucosal epithelia play a similar role during privileged healing in higher vertebrates remains an intriguing question for future research.

In the meanwhile, we are left with the comforting thought that our fishy ancestors, besides endowing us with a spine [7], may have passed down another quite useful invention that prevents us from getting sick after a simple tongue bite.
